# Supplementary material for: COVID-19 mortality in the United States: It’s been two Americas from the start
Source: PLoS One. 2022 Apr 28;17(4):e0265053. doi: 10.1371/journal.pone.0265053 (PMC9049562; doi:10.1371/journal.pone.0265053)
Supplement: Attachment — Submitted filename: Two Americas response to review 3-13-22.docx [file pone.0265053.s001.docx]

Two Americas response to PLOS ONE comments

M.A. Stoto, 3/13/22

*Thank you for the comments we received, and the opportunity to revise and re-submit the paper. Please see our detailed responses below.*

**Journal Requirements:**

When submitting your revision, we need you to address these additional requirements.

1. Please ensure that your manuscript meets PLOS ONE's style requirements, including those for file naming. The PLOS ONE style templates can be found at

<https://journals.plos.org/plosone/s/file?id=wjVg/PLOSOne_formatting_sample_main_body.pdf> and <https://journals.plos.org/plosone/s/file?id=ba62/PLOSOne_formatting_sample_title_authors_affiliations.pdf>

*We have revised the manuscript to meet the journal’s style requirements*

2. PLOS requires an ORCID iD for the corresponding author in Editorial Manager on papers submitted after December 6th, 2016. Please ensure that you have an ORCID iD and that it is validated in Editorial Manager. To do this, go to ‘Update my Information’ (in the upper left-hand corner of the main menu), and click on the Fetch/Validate link next to the ORCID field. This will take you to the ORCID site and allow you to create a new iD or authenticate a pre-existing iD in Editorial Manager. Please see the following video for instructions on linking an ORCID iD to your Editorial Manager account: <https://www.youtube.com/watch?v=_xcclfuvtxQ>

*Done*

3. Please remove your figures from within your manuscript file, leaving only the individual TIFF/EPS image files, uploaded separately. These will be automatically included in the reviewers’ PDF.

*Done*

**Additional Editor Comments:**

1. Please present this manuscript as a brief report rather than full article

*The editorial submission system does not give me this option.*

2. A detailed description on the section of methods is needed.

*The methods section has been expanded as suggested by the Reviewer.*

3. Please carefully revise the manuscript following the reviewer's comments.

*Please see our responses to the specific comments below.*

**Review Comments to the Author (**Reviewer #1)

The authors are to be congratulated for an insightful analysis highlighting marked differences in excess mortality by state. The manuscript is well-written and, although, is short of novelty it does has value in that it re-iterates the need to consider disaggregated data on excess mortality. There are several aspects of the manuscript in its current format that might benefit from further revisions.

*Thank you for these comments. The novelty in this research, we believe, is that it illustrates that regional differences in COVID-19 excess mortality started to mirror deep political differences in the United States long before they became apparent in the uptake of pandemic vaccines. Please see our detailed responses below.*

*Please note that we have also made a number of minor edits, mainly updating references, to reflect new publications and a different situation since we originally submitted this manuscript.*

1. There are statements in the Introduction that require referencing, to add substance to inferences (e.g., last sentence).

*We have added six references to the Introduction, especially the last sentence.*

2. While not a major issue, this Reviewer fails to see the relevance of political context (voting behaviour) for Covid-related excess mortality.

*As noted above, an important contribution of this research is to illustrate that regional differences in COVID-19 excess mortality started to mirror deep political differences in the United States long before they became apparent in the uptake of pandemic vaccines. In trying to maintain the objectivity that is expected in a scientific journal we may have underplayed these issues, so bearing in mind that non-American readers need more information to interpret this, we have added some text to clarify this point.*

3. Might help unfamiliar readers to have more context as to the rationale for regional differences in excess mortality within the US.

*Consistent with our response to comment #2, regional differences in the U.S. are driven in large part by political differences, so we have added text on this too.*

4. In this Reviewer opinion, the Methods section should detail the analytical approach to enable replication of study findings. At a minimum, definition of excess mortality and how differences by region were compared. How did authors adjusted for different region sizes and variation in length of follow-up?

*We have added text to the Methods section on the definition of excess mortality and how it is calculated, how we took into account different region sizes and time periods, and calculated “avoidable mortality.”*

5. Figure are unclear and should include a legend - these should be self-explanatory.

*We have expanded the legends of the figures to make them more self-explanatory.*

6. The research value of the work could be enhanced by discussing potential reasons for variation in excess mortality across the different regions.

*As noted above, we believe that the regional differences are driven by political differences, and have added text to make this point.*
